# Drug Content Uniformity: Quantifying Loratadine in Tablets Using a Created Raman Excipient Spectrum

**DOI:** 10.3390/pharmaceutics13030309

**Published:** 2021-02-27

**Authors:** Amelia Farquharson, Zachery Gladding, Gary Ritchie, Chetan Shende, Joseph Cosgrove, Wayne Smith, Carl Brouillette, Stuart Farquharson

**Affiliations:** 1 Rennselaer Polytechnic Institute, Troy, NY 12180, USA; amy.farquharson@yahoo.com; 2 Real-Time Analyzers, Inc., Middletown, CT 06457, USA; zachary.gladding@gmail.com (Z.G.); chetan@rta.biz (C.S.); carl@rta.biz (C.B.); 3 GER Compliance, Fenton, MO 63026, USA; gary.e.ritchie@gmail.com; 4 Advanced Fuel Research, Inc., East Hartford, CT 06108, USA; cosgrove@afrinc.com; 5 Northrup Grumman, East Hartford, CT 06108, USA; wayne@wsmithconsulting.com

**Keywords:** active pharmaceutical ingredient, drug content uniformity, acceptance value, process control, Raman spectroscopy, loratadine

## Abstract

Raman spectroscopy has proven valuable for determining the composition of manufactured drug products, as well as identifying counterfeit drugs. Here we present a simple method to determine the active pharmaceutical ingredient (API) mass percent in a sample that does not require knowledge of the identities or relative mass percents of the inactive pharmaceutical ingredients (excipients). And further, we demonstrated the ability of the method to pass or fail a manufactured drug product batch based on a calculated acceptance value in accordance with the US Pharmacopeia method for content uniformity. The method was developed by fitting the Raman spectra of 30 Claritin^®^ tablets with weighted percentages of the Raman spectrum of its API, loratadine, and a composite spectrum of the known excipients. The mean loratadine mass of 9.79 ± 40 mg per 100 mg tablet compared favorably to the 10.21 ± 0.63 mg per 100 mg tablet determined using high-performance liquid chromatography, both of which met the acceptance value to pass the 10 mg API product as labelled. The method was then applied to a generic version of the Claritin product that employed different excipients of unknown mass percents. A Raman spectrum representative of all excipients was created by subtracting the API Raman spectrum from the product spectrum. The Raman spectra of the 30 generic tablets were then fit with weighted percents of the pure loratadine spectrum and the created excipient spectrum, and used to determine a mean API mass for the tablets of 10.12 ± 40 mg, again meeting the acceptance value for the 10 mg API product. The data suggest that this simple method could be used to pass or fail manufactured drug product batches in accordance with the US Pharmacopeia method for content uniformity, without knowledge of the excipients.

## 1. Introduction

In the past two decades, Raman spectroscopy has become an important analytical tool for determining the amount of a pharmaceutical active ingredient (API) in solid dose medications [1,2,3,4,5,6,7,8]. Knowledge of the API mass, or mass percentage, is essential in the development and quality control of drug product formulations, as well as in the identification of counterfeit drug products [9,10,11]. The fact that the API mass percent in tablet medications can be determined non-destructively, without sample preparation, and within a few minutes makes Raman spectroscopy ideal for these applications. Consequently, Raman spectroscopy is being used in pharmaceutical company laboratories and production facilities, as well as by enforcement agencies, such as the US Food and Drug Administration [9]. However, making quantitative measurements is challenging. The problem is two-fold. First, the uniformity of the API in a tablet decreases with its mass percent, and second, the size of the focal volume of a typical laser used to generate Raman radiation is several orders of magnitude smaller than the volume of a tablet. Consequently, a single point measurement could miss the API, especially in the case of low dose tablets.

There are a number of publications describing various optical schemes to overcome this under-sampling of the API in such drug products [12,13,14,15,16,17]. The first approach is to simply map the entire surface volume of a tablet, either point-by-point, line scanning, or imaging using back-scattering geometry [6,13,14]. While these approaches generally only measured the surface volume to a depth of 1–2 mm, good quantitative results can be obtained. However, these mapping approaches often take an hour or more. More recently, forward scattering Raman [18], now frequently called transmission Raman [16,17,19], has been developed to measure most of the tablet by collecting the Raman radiation on the tablet side opposite the laser irradiation. While this has proven successful in some cases, it suffers from a decrease in Raman signal intensity by at least an order of magnitude; and, more importantly, scattering due to inactive pharmaceutical ingredients (or excipients), such as diluents and binders, can lead to predicted API mass percents that are incorrect by more than a factor of two [19].

However, in virtually all of these publications, the approach is tailored to a single medication, where model tablets are prepared to mimic products, so that mass percentage plots can be prepared to quantify the API in actual products [7]. This approach may work when the API represents the major portion of the tablet, or only 1 or 2 excipients are present, and known. However, in most tablets, there are at least 4 excipients and sometimes more than 10. Furthermore, the relative excipient mass percentages are often a trade secret, making analysis particularly difficult for forensic samples [20].

Here, we describe a method to overcome this limitation imposed by the excipients by creating a composite Raman spectrum of all of the excipients, which can then be used to quantify the API mass percentage with high accuracy and precision. The method was applied to the determination of the mass percentage of loratadine, the API in the original product Claritin^®^ and the mass percentage of loratadine in an alternative generic product. In addition, the data were compared to high-performance liquid chromatography data, and both data sets were evaluated in terms of acceptance values for passing or failing a manufactured lot, as defined by the US Pharmacopeia (USP) procedure to test dosage content uniformity [21]. Claritin was chosen for this study to represent moderately low dose drugs that require measuring the API mass percent for such USP tests, and not simply the total tablet mass, as is the case for high dose drugs.

## 2. Materials and Methods

### 2.1. Materials

Loratadine, desloratadine, lactose monohydrate, corn starch and magnesium stearate were purchased from USP (Rockville, MD, USA), while acetonitrile, de-ionized (DI) water, phosphoric acid, and potassium dihydrate were purchased from Sigma Aldrich (St. Louis, MO, USA). Reference tablets at 1 cm diameter and ~400 mg, composed of the USP grade Claritin chemical components, loratadine, magnesium stearate, lactose monohydrate and cornstarch, were prepared using a hydraulic press set to 1500 psi. Tablets of Claritin and a generic product (Schering-Plough, Memphis, TN, USA and MSD Consumer Care, Whitehouse Station, NJ, USA, respectively) were purchased from a local pharmacy. Thirty of the Claritin tablets were separately weighed prior to Raman and HPLC measurements.

### 2.2. Method 1: Raman Spectroscopy

All Raman spectroscopic measurements were performed using a LabRaman Analyzer (Real-Time Analyzers, Inc., RTA, Middletown, CT, USA). The analyzer employed a 1064 nm laser that provided a 275 mW, ~100 µm diameter spot, and a diffraction grating—InGaAs array detector combination that provided a 200 to 1800 cm^−1^ spectral region with ~10 cm^−1^ resolution. The tablets were placed in a machined plate, designed to hold various size and shape tablets, and mounted above the laser on an XY positioning stage (Conix Research, Springfield, OR, USA). The stage positioning resolution is ~1 µm, with an XY repeatability of ~20 µm. A 9 × 9 grid covering a 4 × 4 mm^2^ section of one Claritin tablet having a diameter of 6.35 mm and a thickness of 2.44 mm was measured using the XY stage. The laser focal point was set to a depth of ~1 mm below the surface and scanned back and forth over a distance of 3.3 mm for each of the 81 spectra, which consisted of 10 averaged, 4-s integrations (total time at 40 s/spot was ~1 h) [12].

Heat maps, indicating the loratadine mass percent, were prepared for this Claritin tablet using Plotly Chart Studio software (Montreal, Canada). The loratadine mass percentage for this tablet was also determined using S-Quant software (version 1.3.5 RTA) [22]. This and the remaining tablets were each measured as a 3 × 3 grid covering 2 × 2 mm^2^ to a depth of ~1 mm. The 9 spectra, consisting of 10 averaged, 2-s integrations (3 min/tablet), were averaged and then treated using a 3rd-order, 13-point running smooth. The S-Quant software was used to determine the mass percents for all remaining tablets.

### 2.3. Method 2: High-Performance Liquid Chromatography

The HPLC analysis was performed using a Shimadzu LC-10ATvp (Kyoto, Japan) with a D_2_ lamp and a 254 nm detector. A Supelco C8 5 μm 15 cm × 4.6 mm column (Center County, PA, USA) was selected to satisfy the USP L1 phase requirement. A buffer solution was prepared by dissolving 1.75 g potassium dihydrate in 250 mL DI water, which was adjusted to a pH of 3.5 using phosphoric acid [23]. A mobile phase was prepared by adding 150 mL of buffer to 350 mL of acetonitrile, which was sonicated for 20 min and then passed through a 0.45 µm filter. An internal standard was prepared by adding 400 mg of USP desloratadine to 400 mL of the mobile phase, while a calibration series for loratadine was prepared at 0.05, 0.10, 0.15, 0.20, and 0.25 mg/mL in the mobile phase by diluting a 1 mg/mL loratadine reference standard (Sigma Aldrich). Each concentration was measured three times, and the averages were used to prepare the calibration plot. Each Claritin tablet, previously measured by Raman, was weighed, then crushed by mortar and pestle, placed in a 20 mL glass vial, to which 10 mL of the internal standard mobile phase was added. Each sample was vortexed and sonicated to dissolve the tablet. These samples were further dilution by adding 1 mL of the dissolved sample into 9 mL of the internal standard mobile phase to yield an approximate 0.1 mg/mL concentration for both desloratadine and loratadine. These samples were also sonicated for 20 min and passed through 0.45 µm filters.

The column was heated to 35 °C and conditioned first using a 50:50 *v/v* acetonitrile/DI water solution for 30 min, and second with the mobile phase, which was flowed at 0.5 mL/min for 15 min, then 1.0 mL/min for an additional 15 min. For each sample, 20 µL were injected into a loop, 10 µL of which were transferred to the column. All samples were measured in triplicate.

## 3. Results and Discussion

**Figure 1:**
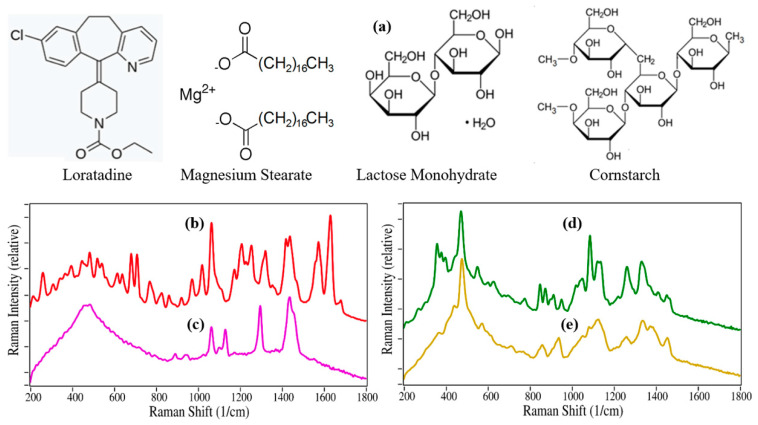
(**a**) Chemical structures for all four Claritin chemicals. Raman spectra of (**b**) loratadine, (**c**) magnesium stearate, (**d**) lactose monohydrate, and (**e**) cornstarch. Sample conditions: Tablets (pressed at 1500 psi). Spectral Conditions: 275 mW at 1064 nm, 10 averaged, 2-s integration scans/spectrum (total time was 200 s).

Commercial 100 mg Claritin tablets are composed of 10 mg loratadine (the antihistamine API), and magnesium stearate, lactose monohydrate, and corn starch at un-labelled mass amounts [24]. The Raman measurement parameters, i.e., laser power, integration time, and spectral averages, were examined to optimize the measurement of loratadine and the Claritin excipients. It was found that 275 mW at 1064 nm, 10 averaged, 2-s integration scans produced quality spectra. In the case of the loratadine, magnesium stearate, lactose monohydrate, and corn starch reference tablets, 100 spectra were averaged to yield high signal-to-noise ratio spectra. These spectra subsequently served as the reference spectra for tablet analysis using the S-Quant software. The Raman spectra for loratadine and magnesium stearate are clearly different than those of lactose monohydrate, and corn starch, however the latter two spectra share several common vibrational modes, as their chemical structures suggest (Figure 1).

**Figure 2:**
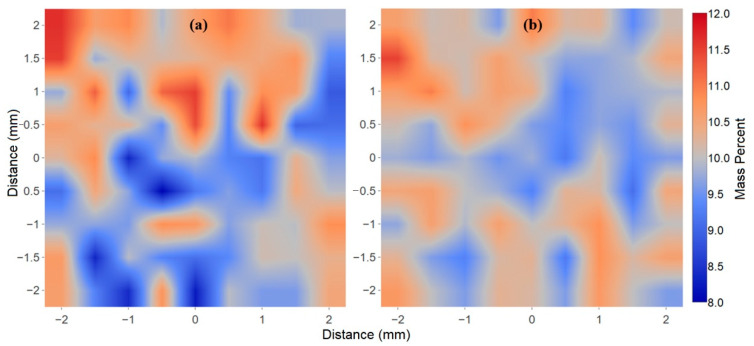
False color heat maps showing the loratadine mass percent (blue = 8%, red = 12%) for a 9 × 9 point grid covering a 4 × 4 mm^2^ surface section to a depth of ~1 mm of a Claritin tablet using 81 Raman spectra determined by (**a**) the 1630 cm^−1^ peak height (baseline corrected) and (**b**) the weighted percentage, 1st-derivative, 200–1800 cm^−1^, spectral fits using pure loratadine, lactose monohydrate and cornstarch. Spectral conditions: 275 mW at 1064 nm, 10 averaged, 4-s integrations/spectrum (total time at 40 s/spot was ~1 h).

An initial Claritin tablet was used to examine the uniform distribution of loratadine in the tablet. This was accomplished by measuring the Raman spectra of 81 discrete points in a 9 × 9 point grid covering a 4 × 4 mm^2^ to a depth of ~1 mm section of the tablet. The loratadine mass percent was calculated using two methods. First, the raw spectra were analyzed by simply measuring the baseline corrected 1630 cm^−1^ carbonyl peak height. The height for each tablet was divided by the average height from all the tablets multiplied by 10, the expected mass percentage. Second, the 1st-derivative, smoothed spectra from 200 to 1800 cm^−1^ were fit with weighted percentages of pure loratadine, magnesium stearate, corn starch and lactose monohydrate, the total of which was set to 100% (Figure S1a). The 1st-derivative was used to remove the effects of baseline offset and slope. The software indicated that magnesium stearate ranged from −2 to +2%, which was not surprising, as this ingredient is used as a mold release agent typically at ~1% [25]. Consequently, magnesium stearate was excluded from further analysis. Not unexpectedly, the peak height method yielded somewhat more scatter in the loratadine mass percent than the full spectrum fitting method. The former ranged from 8.0 to 11.6 mass percent, while the latter ranged from 9.2 to 11.5 mass percent, both indicating uniform distribution of this API throughout the tablet. To further illustrate the API distribution, the loratadine mass percentages for both methods were plotted as 4 mm × 4 mm “heat maps”, in which red was set to 12.0 mass percent and blue to 8.0 mass percent (Figure 2). Overall, these high color-contrast maps show similar distribution of the loratadine, with the highest mass percent in the upper left corner, and lower mass percents in the middle. It is worth noting that the spectral fit method yielded lactose monohydrate and cornstarch mass percentages of 74.4 and 15.6% for the 100 mg tablet. While the excipient mass percentages are not given on the product label, they are likely intended to be 75 and 15 mass percent, since the Summary of Product Characteristics for a generic loratadine product sold by Actavis UK Ltd. (Devon, UK) lists the former as 75 mg per 100 mg tablet [26].

Since it is critical to provide the public with medications that contain the label specified API dosage, the US Pharmacopeia developed a method for pharmaceutical companies to calculate an acceptance value, AV, which indicates the API content uniformity of a production batch and if it passes, i.e., can be released to the public [21]. The AV is calculated in terms of the mean of the API contents, Xbar, expressed as a percentage of the label claim, k, an acceptance constant (k = 2.4 for 10 samples, k= 2.0 for 30 samples), and the sample standard deviation, s, for three conditional cases based on the value of the mean, Xbar, according to the following Equations (1)–(3):

AV = ks, if 98.5% ≤ Xbar ≤ 101.5%,(1)

AV = 98.5 − Xbar + ks, if Xbar < 98.5%,(2)

AV = Xbar − 101.5 + ks, if Xbar > 101.5%.(3)

In general, 10 samples from a batch are analyzed to determine if the API is within 15% of the label claimed dosage. If the calculated AV is modestly greater than 15%, then an additional 20 samples are analyzed. This procedure was followed first for 10, then 30 total Claritin tablets. As described above, each tablet was measured for ~3 min (200 s) using 275 mW at 1064 nm. The loratadine mass percentage for each tablet was calculated by fitting 1st-derivative Raman spectrum of each tablet with the pure spectra of the loratadine, lactose monohydrate, and cornstarch 1500 psi tablets using the S-Quant software (Figure S1a). It was found that the first 10 tablets yielded a mean value of 9.72 mass percent per tablet or 97.2% of the loratadine label claim. Using Equation (2), an AV of 11.9% was obtained, which, being less than 15%, indicates the batch passes. The same procedure was followed for all 30 tablets, yielding 97.0% of the loratadine label claim, and an AV of 9.3%, again passing the batch (Table 1 and Table S1). Even though only 9 points were measured per tablet, it appears to be a good compromise in terms of determining the fate of the batch and the time required to make the measurement. It is worth noting that calculating the loratadine mass percentage for the same 30 tablets using the tablet-to-pure 1500 psi loratadine tablet ratio of the 1630 cm^−1^ Raman peak heights, baseline offset and tilt corrected, yielded 7.75 mg per tablet, with an AV of 31.6, indicating that the batch should be rejected (Table 1, note that the calculated 10-tablet AV using peak heights was worse).

**Figure 3:**
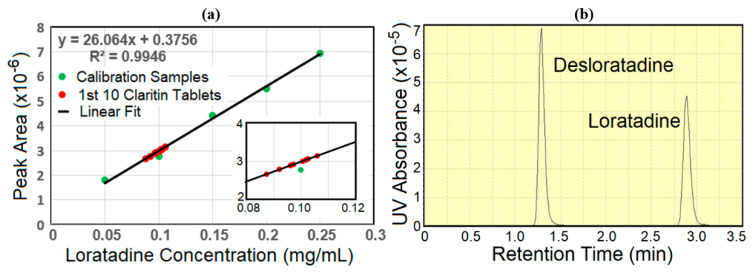
(**a**) HPLC-generated concentration curve for loratadine with calculated concentrations for the 1st 10 Claritin tablets (inset-expanded view of 10 tablets). (**b**) Example chromatograph with desloratadine and Claritin tablet elution peaks. Desloratadine and loratadine peak areas are 2,747,919 and 2,483,413, respectively.

The same 30 tablets were then individually analyzed by high-performance liquid chromatography, the standard method for determining acceptance values for drugs [27,28,29]. Since these measurements employed manual sample introduction into the injection loop, all samples were prepared with 0.1 mg/mL desloratadine as an internal concentration standard. The standard was measured 10 times on the two days that HPLC was performed, and the averaged integrated peak areas were used to correct the loratadine peak areas. Prior to analysis, a calibration curve was developed by measuring loratadine concentrations ranging from 0.05 to 0.25 mg/mL. The loratadine concentration standards, measured in triplicate, produced excellent concentration-to-peak area linearity over the desired concentration range (Figure 3a). For each sample, three 10-µL injections, which produced desloratadine and loratadine peaks with retention times at 1.26 and 2.77 min with peak heights of ~7 × 10^−5^ and 4.5 × 10^−5^ absorbance units, respectively, were averaged to calculate their concentrations. The values were also corrected by the measured mass of each tablet.

As before, the acceptance values were calculated for the first 10 tablets, then all 30 tablets. Analysis of the first 10 tablets yielded a mean value of 9.87 mg per tablet or 98.7% of the loratadine label amount. Therefore, Equation (1) was used (Table 1). An AV of 13.2% was obtained, less than 15%, passing the batch. In the case of all 30 tablets, the mean percent of the label amount was 101.6%, so Equation (3) was used, which yielded an AV of 14.1%, just below 15%, but still passing the batch.

**Figure 4:**
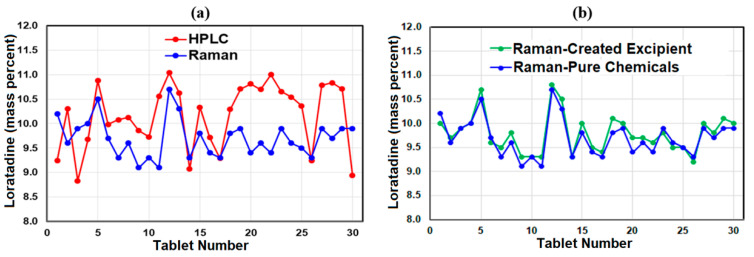
Plots of loratadine mass percentage as a function of tablet number for (**a**) HPLC and Raman using all excipients, and (**b**) Raman using all excipients and a created composite excipient.

As indicated by these analyses, both methods gave mean tablet loratadine mass percentages close to 10% of the total mass, or 10 mg for the 100 mg tablets, and both methods passed the batch based on measuring 10 and 30 tablets. However, the percentage coefficient of variation for the HPLC 30 tablet data was significantly greater than the Raman 30 tablet data using the created excipient spectrum, viz: 6.2% versus 4.1% (Table S1). It is also interesting to note that tablet 12 produced the highest mass percent by both techniques, 11.0 and 10.7 mass percent for HPLC and Raman, respectively. However, there was no such correspondence for the lowest values; Tablet 3 at 8.8 mass percent for HPLC and Tablet 11 at 9.1 mass percent for Raman and (Figure 4a).

**Figure 5:**
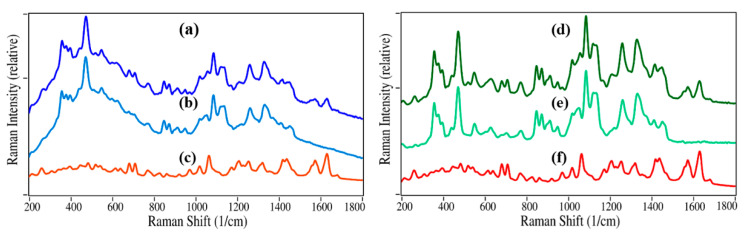
Raman spectra of (**a**) Claritin (30 tablet average), (**b**) the excipient created by subtracting (**c**) loratadine from (**a**,**d**) a generic for Claritin (30 tablet average), (**e**) the excipient created by subtracting (**f**) loratadine from (**d**). Spectral Conditions: 275 mW at 1064 nm, 10 averaged, 2-s integration scans. Spectra are offset for clarity. Intensities are the same scale, except loratadine spectra (**c**) and (**f**), which were increased by a factor of 2.

While the above data suggest that Raman spectroscopy is as good as, or better than, HPLC for determining API mass percents to qualify batches, it required the Raman spectra of the excipients, which may be difficult to obtain or are unknown. These limitations can be overcome by creating a composite excipient spectrum by simply subtracting the spectrum of pure loratadine from the 10 and 30 tablet averaged spectra, respectively (Figure 5a). For this case, the S-Quant software was used to determine the loratadine mass percent for the 10 and 30 tablets using weighted percentages of the pure loratadine 1st-derivative Raman spectrum and the corresponding composite excipient spectrum (Figure S1b).

Again, the acceptance values were calculated for the first 10 tablets, then all 30 tablets. Analysis of the first 10 and 30 tablets yielded mean percentages of 97.8 and 97.9% of the loratadine label amount, respectively. Since these values were both less than 98.5%, Equation (2) was used to calculate their respective AVs, which were 10.6 and 8.7%, respectively, both less than 15%, passing the batches. The created excipient spectrum AV values are slightly better than those obtained using lactose monohydrate and cornstarch pure chemical spectra (Table 1). This improvement is attributed to using one less variable, i.e., one created excipient spectrum instead of two chemical spectra. It is also worth noting that the individual calculated mass percentages for the 30 tablets are nearly identical, with a percentage coefficient of variation only 0.07% higher than the lactose monohydrate and cornstarch-based mass percentages (Figure 4b, Table S1).

The use of a created excipient to determine the API mass percentage for a drug product was taken one step further. As stated in the introduction, many drugs use multiple excipients, and their relative mass percentages may be a trade secret. While this may not be a matter for the original manufacturer, it may be important in forensic studies or identifying counterfeit drugs. To demonstrate this approach, the Raman spectra for a batch of thirty 10 mg per 100 mg loratadine product tablets, as a generic for Claritin, were measured. Then, the pure loratadine spectrum was subtracted from first 10 then 30 tablet averaged spectra to create excipient spectra (Figure 5). The excipient spectra for the generic are considerable different than the Claritin-generated excipient spectra (compare Figure 5b to Figure 5e).

Once again, the acceptance values were calculated for the first 10 tablets, then all 30 tablets. Analysis of the first 10 and 30 tablets yielded mean percentages of 102.5% and 101.2% of the loratadine label amount, respectively. The AVs were then calculated according to Equations (3) and (1), to yield 10.3 and 8.0%, respectively, both less than 15%, and both batches passed (Table 1 and Table S1). These values are slightly better than those obtained using lactose monohydrate and cornstarch spectra.

It is worth noting that the Raman-based created excipient method required less than 5 hr: ~1 hr to prepare the 1500 psi loratadine tablet, ~1.5 hr to measure the loratadine and 30 Claritin tablets at 3 × 3 points each, ~1 hr to fit the spectra and determine the loratadine mass percent for each tablet, and ~1 hr for the USP analysis. In contrast, the HPLC method required more than 13 hr: ~1 hr to prepare buffer solutions and carrier solvents, ~1 h to prepare the reference solution and the calibration standards, ~2 hr to weigh and prepare the 30 individual sample solutions, ~2 hr to pre-treat the column, ~4 hr to measure the 15 calibration and 30 Claritin samples, ~2 hr to determine the loratadine mass percent for each tablet, and ~1 hr for the USP analysis. In addition to analysis time, the development of the Raman method for a new drug only requires preparing and measuring a pressed tablet of the target API, and establishing the best spectral region to use, whereas, the development of an HPLC method requires selecting a column, appropriate buffers, solvents, a reference material, and the measurement conditions. The former can be accomplished in 1 day, while they latter typically takes a week or more [29]. It is of course realized that the Raman method relies on uniform distribution of the API in the tablet, and may be limited to relatively thin tablets, such as those less than 3 mm thick. The analyses of the Claritin and generic tablets clearly indicate that such uniformity exists in these tablets, and likely in similar products.

## 4. Conclusions

Here, we demonstrated a method to determine the API mass percent of a drug product without knowing the excipients, by subtracting the API Raman spectrum from the product Raman spectrum to create a composite excipient spectrum that could be used in mass percentage calculations. The method proved equivalent to the standard HPLC method, at least for Claritin tablets. We believe that the method could be used to (1) qualify content uniformity of a manufactured drug product batch in accordance with the prescribed USP method, even if the excipients are unknown; and (2) identify counterfeit drugs without needing to know the identity or concentration of excipients of the purported drug product.

## Figures and Tables

**Table 1:** Calculated Acceptance Values for 10 and 30 tablets each of Claritin and a generic product based on loratadine mass percents by HPLC, Raman chemical component analysis, and Raman created composite excipient analysis.

Loratadine	Number of Samples, n	Mean, X-bar	Acceptance Constant, k	Standard Deviation, s	EquationUsed	Acceptance Value, AV	Pass? (<15%?)
**Claritin**							
Raman Pure Chemicals	10	97.2	2.4	4.4	2	11.9	Yes
Raman Pure Chemicals	30	97.0	2.0	3.9	2	9.3	Yes
Raman 1630 cm^−1^ Peak Height	10	77.3	2.4	6.8	2	37.6	No
Raman 1630 cm^−1^ Peak Height	30	77.5	2.0	5.3	2	31.6	No
HPLC	10	98.7	2.4	5.6	1	13.5	Yes
HPLC	30	102.1	2.0	6.3	3	13.2	Yes
Raman Created Excipient	10	97.8	2.4	4.1	2	10.6	Yes
Raman Created Excipient	30	97.9	2.0	4.0	2	8.7	Yes
**Generic**							
Raman Created Excipient	10	102.5	2.4	3.9	3	10.3	Yes
Raman Created Excipient	30	101.2	2.0	4.0	1	8.0	Yes

## Data Availability

Not applicable.

## Supplementary Materials

The following are available online at https://www.mdpi.com/1999-4923/13/3/309/s1, Figure S1: Images of S-quant software used to fit the 1st-derivative Raman spectrum of (**a**) a single Claritin tablet with loratadine, corn starch, and lactose monohydrate 1st-derivative Raman spectra, and (**b**) the 1st-derivative Raman spectrum of a single generic tablet with loratadine and a created excipient 1st-derivative Raman spectra. Measured Raman spectra are shown for clearer presentation, Table S1: Calculated loratadine mass percents with statistics for 1st 10 and all 30 Claritin and a generic tablets by HPLC, Raman chemical component analysis, Raman created excipient analysis, and 1630 cm^−1^ peak height analysis.
