# Supplementary material for: Drug Content Uniformity: Quantifying Loratadine in Tablets Using a Created Raman Excipient Spectrum
Source: Pharmaceutics. 2021 Feb 27;13(3):309. doi: 10.3390/pharmaceutics13030309 (PMC7997337; doi:10.3390/pharmaceutics13030309)
Supplement: Supplementary file 1 [file pharmaceutics-13-00309-s001.pdf]

pharmaceutics

Supplementary Materials: Drug Content Uniformity: Quantifying Loratadine in Tablets Using a Created Raman Excipient Spectrum

Amelia Farquharson, Zachery Gladding, Gary Ritchie, Chetan Shende, Joseph Cosgrove, Wayne Smith, Carl Brouillette and Stuart Farquharson

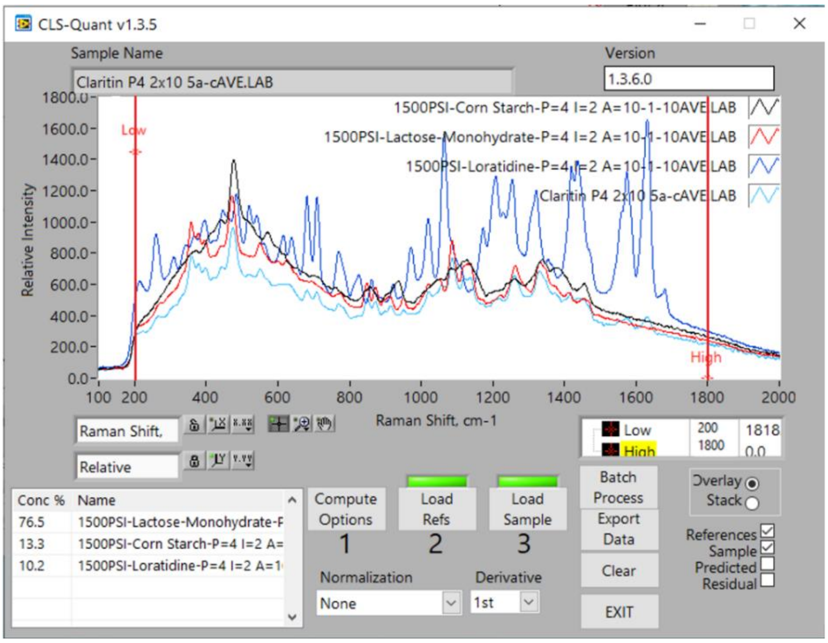

(**a**)

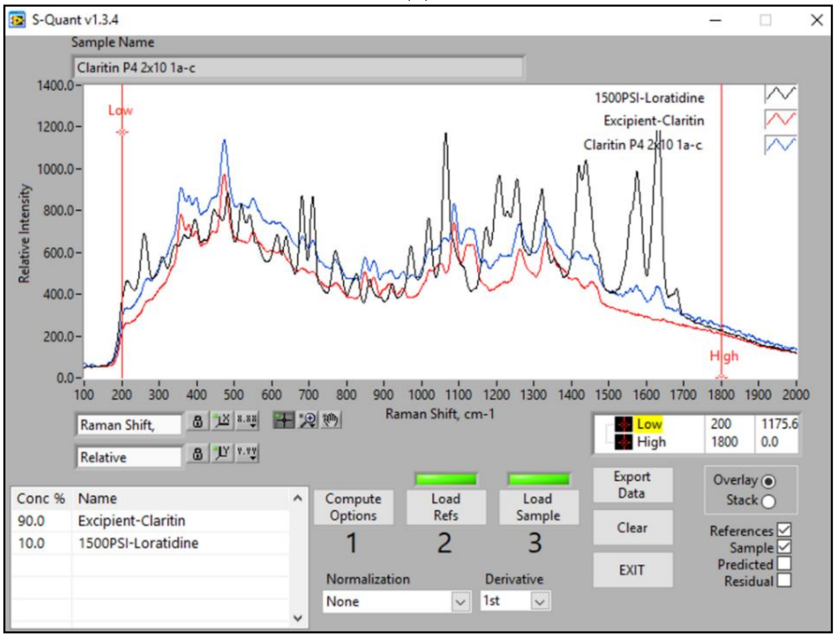

(**b**)

Figure S1. Images of S-quant software used to fit the 1st-derivative Raman spectrum of (**a**) a single Claritin tablet with loratadine, corn starch, and lactose monohydrate 1st-derivative Raman spectra, and (**b**) the 1st-derivative Raman spectrum of a single generic tablet with loratadine and a created excipient 1st-derivative Raman spectra. Measured Raman spectra are shown for clearer presentation.

Table S1. Calculated loratadine mass percents with statistics for 1st 10 and all 30 Claritin and a generic tablets by HPLC, Raman chemical component analysis, Raman created excipient analysis, and 1630 cm⁻¹ peak height analysis.

	HPLC	Raman Spectroscopy						
		Claritin					Generic	Claritin
		Pure Chemical Analysis			Created Excipient Analysis			1630 cm^{-1} Peak
**Tablet Number**	**% Loratadine**	**% Loratadine**	**% Lactose Monohydrate**	**% Cornstarch**	**% Loratadine**	**% Excipient**	**% Loratadine**	**% Loratadine**
1	9.2	10.2	76.2	13.6	10.0	90.0	10.70	8.36
2	10.3	9.6	72.1	18.4	9.7	90.3	10.70	7.99
3	8.8	9.9	73.7	16.3	9.9	90.1	10.50	7.25
4	9.7	10.0	73.3	16.7	10.0	90.0	10.10	7.99
5	10.9	10.5	73	16.5	10.7	89.3	10.10	8.09
6	10.0	9.7	76.2	14.1	9.6	90.4	9.50	7.43
7	10.1	9.3	71.5	19.2	9.5	90.5	10.50	6.78
8	10.1	9.6	72.5	17.9	9.8	90.2	10.20	8.83
9	9.9	9.1	73.4	17.5	9.3	90.7	9.80	6.69
10	9.7	9.3	75.3	15.4	9.3	90.7	10.40	7.90
11	10.6	9.1	71.9	19	9.3	90.7	10.10	7.90
12	11.0	10.7	74.1	15.2	10.8	89.2	10.00	8.18
13	10.6	10.3	73.6	16.1	10.5	89.5	9.60	7.99
14	9.1	9.3	74.3	16.5	9.3	90.7	9.90	7.62
15	10.3	9.8	72.7	17.5	10.0	90.0	10.20	8.18
16	9.7	9.4	74.3	16.2	9.5	90.5	10.00	7.90
17	9.3	9.3	74.4	16.2	9.4	90.6	9.90	7.25
18	10.3	9.8	72	18.2	10.1	89.9	9.70	8.36
19	10.7	9.9	74.8	15.3	10.0	90.0	9.80	7.62
20	10.8	9.4	71.6	19	9.7	90.3	10.10	6.51
21	10.7	9.6	73.3	17.1	9.7	90.3	9.60	7.25
22	11.0	9.4	73.2	17.4	9.6	90.4	9.60	7.25
23	10.7	9.9	75.2	14.9	9.8	90.2	10.00	7.90
24	10.5	9.6	75.3	15.1	9.5	90.5	10.60	8.09
25	10.4	9.5	73.9	16.6	9.5	90.5	9.90	7.71
26	9.2	9.3	75.9	14.8	9.2	90.8	10.50	8.27
27	10.8	9.9	73.9	16.1	10.0	90.0	10.80	7.25
28	10.8	9.7	73.1	17.3	9.8	90.2	11.00	7.90
29	10.7	9.9	72	18.2	10.1	89.9	10.20	7.99
30	8.9	9.9	73.4	16.6	10.0	90.0	9.70	7.99
**10 Tablet Mean**	9.87	9.72	73.68	16.63	9.78	90.22	10.25	7.73
**Std Dev**	0.56	0.44	1.36	1.48	0.41	0.41	0.39	0.68
**% Coef Var**	**5.70%**	**4.49%**	1.85%	8.87%	**4.22%**	0.46%	**3.80%**	**8.83%**
**30 Tablet Mean**	10.21	9.70	73.54	16.69	9.79	90.21	10.12	7.75
**Std Dev**	0.630	0.393	1.365	1.476	0.403	0.403	0.399	0.531
**% Coef Var**	**6.17%**	**4.05%**	1.86%	8.84%	**4.12%**	0.45%	**3.94%**	**6.85%**
